# Teriflunomide Preserves Neuronal Activity and Protects Mitochondria in Brain Slices Exposed to Oxidative Stress

**DOI:** 10.3390/ijms23031538

**Published:** 2022-01-28

**Authors:** Bimala Malla, Agustin Liotta, Helena Bros, Rebecca Ulshöfer, Friedemann Paul, Anja E. Hauser, Raluca Niesner, Carmen Infante-Duarte

**Affiliations:** 1 Institute for Medical Immunology, Charité—Universitätsmedizin Berlin, Corporate Member of Freie Universität Berlin, Humboldt-Universität zu Berlin, Augustenburger Platz 1, 13353 Berlin, Germany; bimala.malla@charite.de (B.M.); hnbros@gmail.com (H.B.); rebecca.ulshofer@charite.de (R.U.); 2 Klinik für Anästhesiologie mit Schwerpunkt Operative Intensivmedizin, Charité—Universitätsmedizin Berlin, Charitéplatz 1, 10117 Berlin, Germany; agustin.liotta@charite.de; 3 Experimental and Clinical Research Center (ECRC), MDC for Molecular Medicine and Charité—Universitätsmedizin Berlin, Lindenberger Weg 80, 13125 Berlin, Germany; friedemann.paul@charite.de; 4 NeuroCure Clinical Research Center, Charité—Universitätsmedizin Berlin, Charitéplatz 1, 10117 Berlin, Germany; 5 Deutsches Rheuma-Forschungszentrum, Charitéplatz 1, 10117 Berlin, Germany; hauser@drfz.de (A.E.H.); niesner@drfz.de (R.N.); 6 Medizinische Klinik mit Schwerpunkt Rheumatologie und Klinische Immunologie, Charité—Universitätsmedizin Berlin, Corporate Member of Freie Universität Berlin, Humboldt-Universität zu Berlin, Charitéplatz 1, 10117 Berlin, Germany; 7 Dynamic and Functional In Vivo Imaging, Veterinary Medicine, Freie Universität Berlin, 14163 Berlin, Germany

**Keywords:** mitochondria, neurodegeneration, teriflunomide (TFN), oxidative stress, dihydroorotate dehydrogenase (DHODH), multiple sclerosis, mitochondrial morphology, mitochondrial motility, acute hippocampal slices, two-photon microscopy

## Abstract

Teriflunomide (TFN) limits relapses in relapsing–remitting multiple sclerosis (RRMS) by reducing lymphocytic proliferation through the inhibition of the mitochondrial enzyme dihydroorotate dehydrogenase (DHODH) and the subsequent modulation of de novo pyrimidine synthesis. Alterations of mitochondrial function as a consequence of oxidative stress have been reported during neuroinflammation. Previously, we showed that TFN prevents alterations of mitochondrial motility caused by oxidative stress in peripheral axons. Here, we aimed to validate TFN effects on mitochondria and neuronal activity in hippocampal brain slices, in which cellular distribution and synaptic circuits are largely preserved. TFN effects on metabolism and neuronal activity were investigated by assessing oxygen partial pressure and local field potential in acute slices. Additionally, we imaged mitochondria in brain slices from the transgenic Thy1-CFP/COX8A)S2Lich/J (mitoCFP) mice using two-photon microscopy. Although TFN could not prevent oxidative stress-related depletion of ATP, it preserved oxygen consumption and neuronal activity in CNS tissue during oxidative stress. Furthermore, TFN prevented mitochondrial shortening and fragmentation of puncta-shaped and network mitochondria during oxidative stress. Regarding motility, TFN accentuated the decrease in mitochondrial displacement and increase in speed observed during oxidative stress. Importantly, these effects were not associated with neuronal viability and did not lead to axonal damage. In conclusion, during conditions of oxidative stress, TFN preserves the functionality of neurons and prevents morphological and motility alterations of mitochondria.

## 1. Introduction

Multiple sclerosis (MS) is a chronic inflammatory and neurodegenerative disease that represents one of the most common non-traumatic incapacitating neurological diseases in young adults [1]. Pathologically, MS is characterized by inflammation, demyelination, and neuroaxonal damage [1,2]. The MS course is generally characterized by an initial relapsing–remitting MS (RRMS) phase, in which acute attacks are followed by complete or partial recovery. Around 80–85% of the RRMS patients develops a secondary progressive MS (SPMS) within 20–30 years after diagnosis, which is characterized by the progressive accumulation of disability, generally independent of relapses [1,3]. About 10–15% of patients manifest a primary progressive MS [PPMS] characterized by chronic progression from the onset of the disease.

Although the exact disease pathogenesis remains unclear, it is assumed that an autoimmune attack mediated by autoreactive lymphocytes initiates the disease and leads to neurodegenerative processes [4,5]. In this context, oxidative stress caused by the sustained release of reactive oxygen and nitrogen species (ROS and NOS) by inflammatory cells appears to be implicated in the damaging cascade inside the CNS [6,7,8,9]. Numerous studies indicated that oxidative stress and mitochondria are the key players in neurodegeneration [9,10,11]. Moreover, mitochondrial alterations are considered one of the early contributors of the disease that leads to virtual hypoxia, causing energy deficiency, ionic imbalance, as well as tissue damage [12,13,14]. Hence, mitochondria could represent a target for therapies [4,9,15,16,17,18].

Teriflunomide (TFN) is a disease-modifying drug (DMT) that has been recently approved as an oral therapy for patients with RRMS [19,20]. TFN is the active metabolite of leflunomide, which has been widely used to treat rheumatic arthritis [21] and is known to exert its effect by non-competitively and reversibly inhibiting dihydroorotate dehydrogenase (DHODH). DHODH is a mitochondrial enzyme located on the inner mitochondrial membrane that is involved in de novo pyrimidine synthesis [19,20,22,23]. DHODH-inhibition, therefore, primarily leads to the regulation of rapidly dividing cells, reducing B and T cell proliferation and modulating inflammation in MS [22,24,25]. Due to its immunomodulatory potential, TFN has been implicated in RRMS.

Since TFN inhibits an enzyme that is closely associated with mitochondrial biology via the respiratory chain, we hypothesized that treatment of MS patients with TFN may also affect neuronal mitochondria. Although in MS, autoimmune attack is assumed to be one of the key players leading to neurodegenerative processes [26], numerous studies indicate that inflammation-induced oxidative stress in CNS leads to mitochondrial damage and consequently, to neurodegeneration [9,10,11,27].

Recently, we showed that TFN could prevent alterations in mitochondrial morphology, motility, as well as oxidation potential in peripheral spinal root explants during oxidative stress [28]. Considering the central role of inflammation-mediated oxidative stress and subsequent mitochondria damage in MS, we aimed here to determine whether TFN may also affect mitochondrial functionality and dynamics in hippocampal brain slices, which retain the complexity and physiology of central nervous system (CNS) structures and were exposed to oxidative stress conditions [29]. Thus, we have investigated the effect of TFN on synaptic transmission, metabolism, as well as on the dynamics of CNS mitochondria in a model of murine acute hippocampal slices exposed to exogenous hydrogen peroxide (H_2_O_2_) that induces oxidative stress in CNS cells.

## 2. Results

### 2.1. TFN Restores Tissue Respiration during Oxidative Stress

**Figure 1:**
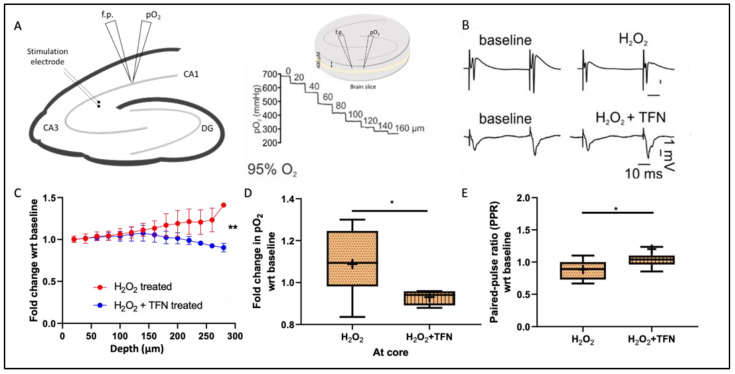
Electrophysiology recordings of pO_2_ and local field potential in acute hippocampal slice: (**A**) Left, schematic representation of hippocampal slice and the placement of stimulation, recording, and pO_2_ electrodes. The Schaffer collaterals were electrically stimulated in the stratum radiatum of the cornu ammonis (CA) and the O_2_ recording and population spikes (PS) were simultaneously obtained between CA3 and CA1 in close vicinity. Middle upper, representation of 400 µM-thick brain slice in interface chamber which is supplied with carbogen from surface and bottom. The pO_2_ decreases with each step inside the tissue up to the core (not shown in the figure), after which it starts increasing (due to the distance from the source of oxygen, i.e., from the surface and the bottom) providing a typical depth profile, as shown in middle lower figure—the pO_2_ measurement steps at the interval of 20 µm from the surface to the core; (**B**) a typical population spike in a paired pulse at baseline, with H_2_O_2_ and H_2_O_2_ + TFN treatment; (**C**) fold change in the pO_2_ at tissue depth in the interval of 20 µm with respect to the baseline; (**D**) fold change in pO_2_ at the core with respect to the baseline recording; (**E**) the paired-pulse ratio (PPR) of PS2 and PS1 with respect to the respective baseline recordings between H_2_O_2_ treatment and H_2_O_2_ + TFN treatment. Graphs are shown in Tukey boxplots. Inside the box, ‘+’ delineates the mean. * *p* ≤ 0.05, ** *p* ≤ 0.01.

To understand the effects of TFN on the metabolic activity in the nervous tissue, we investigated changes in oxygen partial pressure (pO_2_) levels in hippocampal slices at baseline and after treatments with H_2_O_2_ (200 µM) and H_2_O_2_ (200 µM) + TFN (50 µM). In vitro, changes in pO_2_ correlates inversely with neuronal oxygen consumption and cellular metabolism due to the constant supply with oxygen and glucose. To determine pO_2_, we performed a gradient descent of the oxygen electrode into the slice, which we refer to as ‘steps’ from the surface with the increment of 20 µm up to the core of the slice. A total of 10 slices were analyzed in three independent experiments. As depicted in Figure 1C, the core was located between the depth of 240 and 280 µm, after which the pO_2_ level no longer decreased (see methods). Thus, the depicted data covers only the descent to the core. Then, the oxygen electrode was raised to a 100 µm depth from the surface of the slice and 15 min of baseline activity was recorded. Immediately following the baseline recording, the H_2_O_2_ or H_2_O_2_ + TFN treatment in an artificial cerebrospinal fluid (aCSF) was applied for 30 min. Again, the steps were performed from the surface of the slice towards the core.

Figure 1A shows the depth profile of the O_2_ steps with the corresponding partial pressures of oxygen. At the slice surface, pO_2_ of ~680 mmHg corresponded to carbogen. Figure 1C,D shows the fold change in the pO_2_ with respect to the baseline measurement. We observed that the pO_2_ increased near to the core, pointing to an increase in pO_2_ due to a reduced oxygen consumption in H_2_O_2_-stressed tissue (1.09 ± 0.16-fold (mean ± SD) relative the baseline). In contrast, the pO_2_ decreased towards the core when TFN treatment was added (H_2_O_2_ + TFN; 0.93 ± 0.04 fold (mean ± SD) relative to untreated), indicating an increase in the consumption of oxygen in the presence of TFN with respect to H_2_O_2_ treatment alone (Figure 1D,E).

### 2.2. TFN Prevents Oxidative Stress-Mediated Depression of Synaptic Transmission

Furthermore, we investigated the effect of oxidative stress and TFN application on synaptic transmission in acute hippocampal tissue. For that, stimulus-induced local field potential (LFP) signals were recorded in CA1 while stimulating the Schaffer collaterals using a paired-pulse facilitation protocol (see methods). The amplitudes of the population spike responses during baseline and treatment with H_2_O_2_ (200 µM) or H_2_O_2_ (200 µM) + TFN (50 µM) were measured.

Figure 1B shows a typical population spike (PS) in response to a paired-pulse stimulation. The amplitudes of the population spikes were measured by subtracting the maxima and minima of the spike. Then, we compared the short-term facilitation/paired-pulse ratio (PPR), which is the ratio of the amplitude of the second population spike (PS2) to the first population spike (PS1) and normalized to the baseline recordings. We observed a depression of PPR in H_2_O_2_-stressed brain slices treatment (0.88 ± 0.14-fold (mean ± SD) relative to the baseline), while the presence of TFN increased the PPR (H_2_O_2_ + TFN treatment (1.2 ± 0.62-fold (mean ± SD) relative to the baseline) (Figure 1E).

### 2.3. TFN Does Not Prevent Oxidative Stress-Mediated ATP Decrease in Acute Hippocampal Slices

**Figure 2:**
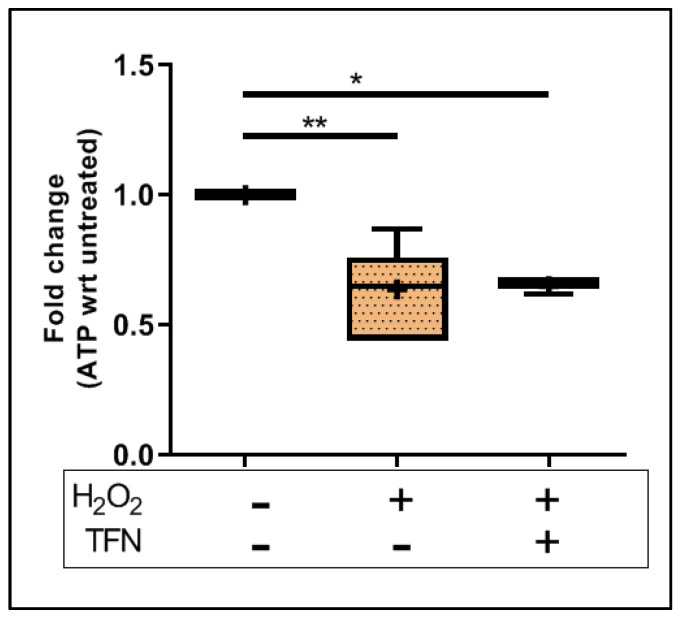
ATP levels in brain tissue exposed to oxidative stress with/out TFN treatment. Graphs are expressed as fold change with respect to the baseline and shown in Tukey boxplots. Inside the box, ‘+’ delineates the mean. * *p* ≤ 0.05 and ** *p* ≤ 0.01.

To understand the effect on the energy metabolism of mitochondria, we investigated the effect of TFN on ATP levels in the acute hippocampal slices exposed to oxidative stress. We observed that the ATP amount in the slices treated with H_2_O_2_ significantly decreased (0.64 ± 0.16-fold (mean ± SD)) with respect to untreated controls. The treatment with TFN along with H_2_O_2_ did not affect or restore the ATP levels (0.65 ± 0.03-fold (mean ± SD)). (Figure 2).

### 2.4. TFN Prevented Oxidative Stress-Induced Decrease in Mitochondrial Area in Acute Hippocampal Slices

We have shown previously that in peripheral nerves, mitochondria exposed to oxidative stress are affected even prior to the axonal damages [28,30] and that TFN prevents these mitochondrial alterations [28]. To monitor how TFN affects changes of mitochondria morphology during oxidative stress within the CNS, hippocampal slices from transgenic Tg(Thy1-CFP/COX8A)S2Lich/J mice (mitoCFP mice) that express cyan fluorescence protein (CFP) in neuronal mitochondria were imaged before (baseline image) and after the treatment with H_2_O_2_ or H_2_O_2_ + TFN. Then, the values of treated slices were normalized with their respective baseline images.

**Figure 3:**
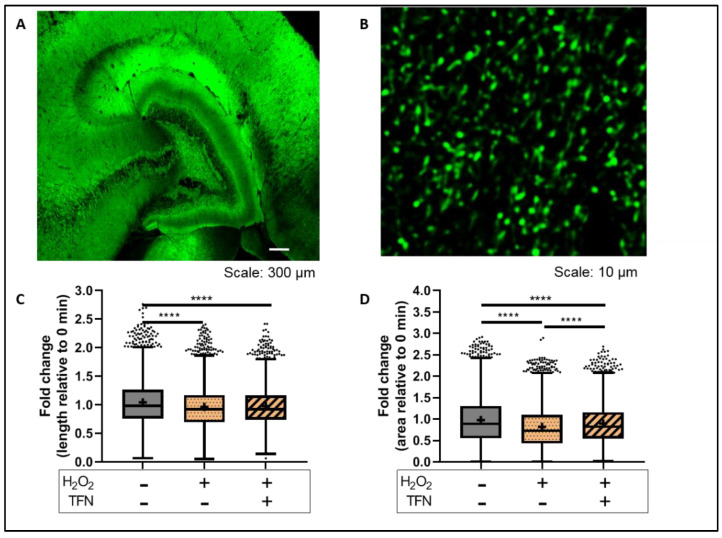
Mitochondrial morphology in brain tissue exposed to oxidative stress with/out TFN: (**A**) Representative confocal image of murine hippocampal section from mitoCFP mice. The fluorescence of cyan fluorescence protein in mitochondria is shown in green; (**B**) representative two-photon microscope image of murine hippocampal section from mitoCFP mice. Mitochondria are shown in green; fold change in (**C**) mitochondrial length and (**D**) mitochondrial area with respect to 0 min slice. Graphs are expressed as fold change with respect to the baseline and shown in Tukey boxplots. Inside the box, ‘+’ delineates the mean. **** *p* ≤ 0.0001.

We observed that H_2_O_2_ reduced mitochondrial length (0.96 ± 0.37-fold (mean ± SD)) and that this effect was not abolished in the presence of TFN (0.97 ± 0.34-fold (mean ± SD)) (Figure 3C). However, the H_2_O_2_-induced reduction of mitochondrial area was partly prevented in presence of TFN treatment (data shown as mean ± SD: untreated: 0.97 ± 0.56-fold; H_2_O_2:_ 0.82 ± 0.48-fold; and H_2_O_2_ ± TFN: 0.89 ± 0.47-fold) (Figure 3D) (Table 1 and Table 2).

### 2.5. TFN Did Not Prevent Oxidative Stress-Mediated Alterations in Mitochondrial Motility in Acute Hippocampal Slices

To monitor effects of TFN on oxidative stress-induced motility alterations, we obtained time-lapse images of the slices before (baseline image) and after the treatment with H_2_O_2_ or H_2_O_2_ + TFN, as mentioned above. Mitochondrial motility-related parameters of treated slices were normalized to their respective baseline images as explained in the methods.

**Figure 4:**
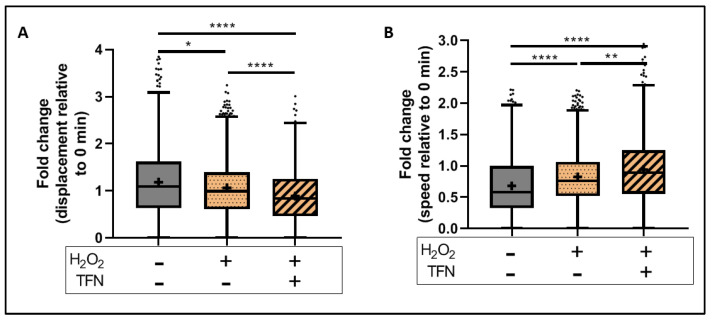
Mitochondrial motility in brain slices exposed to oxidative stress with the presence/absence of TFN: Fold change in (**A**) mitochondrial displacement and (**B**) motility speed with respect to 0 min slice. Graphs are expressed as fold change with respect to the baseline and shown in Tukey boxplots. Inside the box, ‘+’ delineates the mean. ‘−’ horizontally aligned with H_2_O_2_ and TFN denotes no H_2_O_2_ or TFN treatment. * *p* ≤ 0.05, ** *p* ≤ 0.01, and **** *p* ≤ 0.0001.

In our setup, the H_2_O_2_-induced decrease in mitochondrial displacement was further decreased in the presence of TFN (data shown as mean ± SD: H_2_O_2_ treated: 1.06 ± 0.66-fold and H_2_O_2_ + TFN treated: 0.88 ± 0.59-fold) (Figure 4A). Similarly, the H_2_O_2_- induced increase in mitochondrial speed was further increased by TFN treatment (H_2_O_2:_ 0.78 ± 0.40-fold and H_2_O_2_ + TFN: 0.94 ± 0.56-fold) (Figure 4B) (Table 3 and Table 4).

### 2.6. TFN Prevented the Oxidative Stress-Promoted Decrease in Length of Network Mitochondria and Size of Puncta-Shaped and Network Mitochondria in Acute Hippocampal Slices

**Figure 5:**
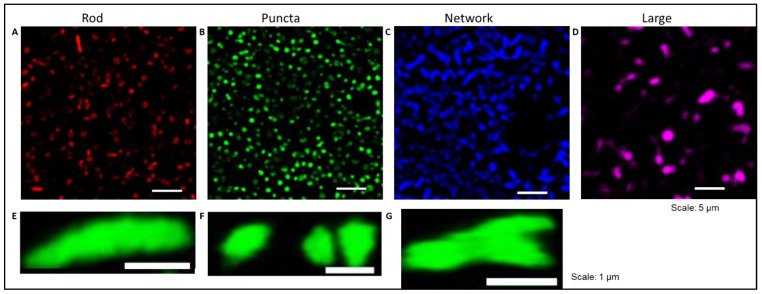
Morphotypes of mitochondria in murine acute hippocampal slices during oxidative stress with/out TFN. Representative segmented image of rod-shaped, puncta-shaped, network, and large mitochondria respectively (**A**–**D**). Representative image of an individual rod (**E**), puncta (**F**), and network (**G**) mitochondria.

We know that mitochondria are dynamic organelles that can change their shape, ranging from punctuate structures to tubular networks depending on the cellular needs. Hence, we processed the images and segmented the fluorescent mitochondria into four different morphological categories: rod-shaped, puncta-shaped, network, and large mitochondria. Morphological parameters of mitochondria in treated slices were renormalized to their respective baseline images (Figure 5).

**Figure 6:**
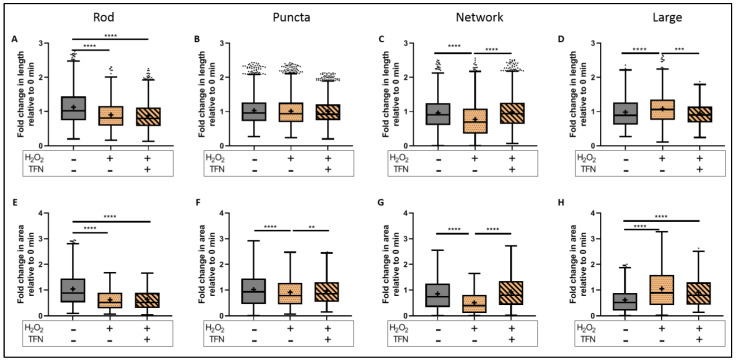
Morphological alterations in different mitochondrial morphotypes during induced oxidative stress with/out TFN. Fold change in length of rod-shaped (**A**), puncta-shaped (**B**), network (**C**), and large mitochondria (**D**) during oxidative stress with/out TFN relative to 0 min slices. Fold change in area of rod-shaped (**E**), puncta-shaped (**F**), network (**G**), and large mitochondria (**H**) during oxidative stress with/out TFN relative to 0 min slices. Graphs are expressed as fold change with respect to the baseline and shown in Tukey boxplots. Inside the box, ‘+’ delineates the mean. ‘−’ horizontally aligned with H_2_O_2_ and TFN denotes no H_2_O_2_ or TFN treatment. ** *p* ≤ 0.01, *** *p* ≤ 0.001, and **** *p* ≤ 0.0001.

To evaluate the morphological parameters of mitochondria in treated slices, data were normalized to their respective baseline images. We showed that oxidative stress reduced the length of rod-shaped, network, and large mitochondria, while the addition of TNF prevented these alterations in network and large types but not in rod-shaped mitochondria (Figure 6A–D) (in rod-shaped mitochondria shown as mean ± SD; untreated: 1.12 ± 0.52 length and 1.04 ± 0.66 area; H_2_O_2_: 0.90 ± 0.41 length and 0.62 ± 0.39 area; and H_2_O_2_ + TFN: 0.88 ± 0.41 length and 0.64 ± 0.40 area) (Table 1 and Table 2).

TFN also prevented H_2_O_2_- induced area reduction in puncta, network, and large (here only a trend) forms (Figure 6E–H). Again, in rod-shaped mitochondria, TFN has no effects on H_2_O_2_-promoted size reduction. In the case of puncta-shaped mitochondria, there was no difference in the length (data shown as mean ± SD: untreated: 1.03 ± 0.43; H_2_O_2_: 1.01 ± 0.43; H_2_O_2_ + TFN: 1.00 ± 0.36); however, mitochondria became smaller (data shown as mean ± SD: untreated: 1.02 ± 0.65; H_2_O_2_: 0.91 ± 0.57) after exposure to H_2_O_2_. TFN- treatment prevented the reduction of mitochondrial size (0.96 ± 0.52). Similarly, TFN- treatment could prevent reduction in length and size of network-shaped mitochondria induced by H_2_O_2_ (data shown as mean ± SD: untreated: 0.96 ± 0.46 length, 0.85 ± 0.60 area; H_2_O_2_: 0.77 ± 0.51 length, 0.51 ± 0.45 area; H_2_O_2_ + TFN: 0.99 ± 0.45 length, 0.93 ± 0.62 area). In contrast, H_2_O_2_ induced the increase in length and size of large mitochondria (data shown as mean ± SD: untreated: 0.98 ± 0.46 and 0.61 ± 0.48 area; H_2_O_2_: 1.08 ± 0.44 length and 1.05 ± 0.76 area). TFN prevented the oxidative stress-related increase in length but not the area of large mitochondria (data shown as mean ± SD: 0.95 ± 0.33 length and 0.91 ± 0.61 area) (Table 1 and Table 2).

### 2.7. TFN Enhanced Mitochondrial Speed in Puncta-Shaped Mitochondria in Acute Hippocampal Slices Exposed to Oxidative Stress

**Figure 7:**
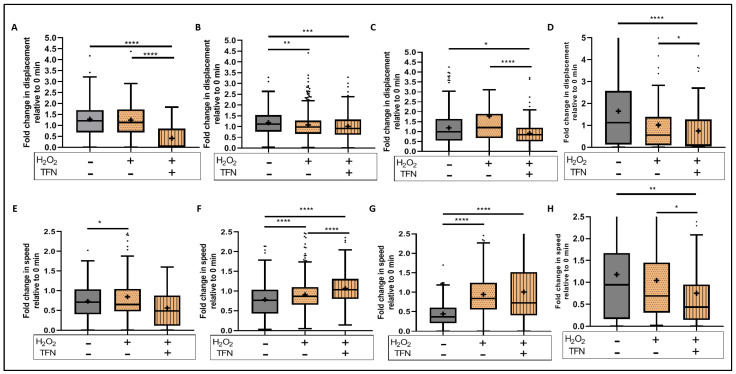
Motility alterations in different mitochondrial morphotypes during induced oxidative stress with/out TFN: Fold change in displacement of rod-shaped (**A**), puncta-shaped (**B**), network (**C**), and large mitochondria (**D**) during oxidative stress with/out TFN relative to 0 min slices. Fold change in transportation speed of rod-shaped (**E**), puncta-shaped (**F**), network (**G**), and large mitochondria (**H**) during oxidative stress with/out TFN relative to 0 min slices. Graphs are expressed as fold change with respect to the baseline and shown in Tukey boxplots. Inside the box, ‘+’ delineates the mean. ‘−’ horizontally aligned with H_2_O_2_ and TFN denotes no H_2_O_2_ or TFN treatment. * *p* ≤ 0.05, ** *p* ≤ 0.01, *** *p* ≤ 0.001, and **** *p* ≤ 0.0001.

Similarly, we investigated the motility parameters using the time-lapse images (Figure 7). We observed that TFN reduced the displacement of all mitochondrial forms as shown in Figure 7A–D (data shown as mean ± SD: untreated: 1.08 ± 0.8 (rod), 1.18 ± 0.55 (puncta), 1.19 ± 0.93 (network), 1.65 ± 1.77 (large); H_2_O_2_ + TFN: 0.42 ± 0.55 (rod), 1.01 ± 0.57 (puncta), 0.91 ± 0.60 (network), and 0.75 ± 1.11 (large)). Additionally, in case of rod-shaped and network mitochondria, it accentuated the effect of H_2_O_2_ (data shown as mean ± SD: 1.08 ± 0.68 (rod), 1.08 ± 0.60 (puncta), 1.78 ± 2.16 (network), and 1.02 ± 1.25 (large)) (Table 3). Furthermore, TFN enhanced the effect of H_2_O_2_, increasing the speed in puncta-shaped and network mitochondria and decreasing speed of the large morphotype (Figure 7E–H) (data shown as mean ± SD: untreated: 0.73 ± 0.38 (rod), 0.78 ± 0.43 (puncta), 0.44 ± 0.31 (network), 1.18 ± 1.29 (large); H_2_O_2_: 0.85 ± 0.60 (rod), 0.91 ± 0.37 (puncta), 0.94 ± 0.65 (network), 1.05 ± 1.03 (large); H_2_O_2_ + TFN: 0.56 ± 0.51 (rod), 1.07 ± 0.39 (puncta), 1.01 ± 0.76 (network), and 0.75 ± 0.91 (large)) (Table 4).

## 3. Discussion

Mitochondrial alteration during inflammation is one of the major factors that contribute to neurodegeneration in inflammatory diseases such as MS. Thus, besides the importance of controlling inflammation, MS treatments should also aim at protecting mitochondria from damage [17]. In this context, we have investigated the therapeutic potential of the DHODH inhibitor TFN, an approved drug for the treatment of RRMS [19,20], on both neuronal and mitochondrial protection. Using a model in peripheral root explants, we have previously shown that mitochondria become shorter and rounder, and were less motile, during oxidative stress [28,30]. Importantly, using this model of peripheral nerves, we recently demonstrated that TFN could prevent the morphologic alterations of axonal mitochondria caused by oxidative stress [28]. To understand the effect of TFN on neuronal activity and neuronal mitochondria within the CNS, we used here acute hippocampal sections from transgenic mice that express CFP in neuronal mitochondria. To induce oxidative stress on neurons, slices were treated with H_2_O_2_, and the effects of TFN on H_2_O_2_-mediated neuronal and mitochondria changes were investigated. In the CNS slice model, TFN was used at a concentration of 50 µM. Using peripheral spinal root explants, we have already previously shown that TFN prevented oxidative stress-induced mitochondrial alterations at 1 µM concentrations [28]. However, since in the CNS sections the TFN dissolved in DMSO had to penetrate to depths of 50–100 µm, 50 µM of TFN was used to ensure sufficient diffusion into the tissue slices.

Our findings with electrophysiological recordings of pO_2_ and LFP revealed that TFN treatment could prevent the reduction of oxygen consumption and synaptic transmission upon the induced oxidative insult. An increment in pO_2_ with respect to the baseline recording during oxidative stress (Figure 1C,D) suggested a decrease in the consumption of oxygen, and hence, slower oxidative metabolism in the tissue. TFN could prevent this effect (Figure 1D), as pO_2_ decrease corresponds to increased oxygen consumption, and hence, suggests a restoration of the metabolic activity.

In addition, in the tissue exposed to oxidative stress we observed a decrement in LFP, clearly signaling the depression of neuronal firing. This is in line with the report of Ohashi et al., 2016, which showed H_2_O_2_-induced reduction in neuronal excitability in ventral horn neurons [31]. The depression in neuronal firing was prevented by TFN (Figure 1E). Importantly, inhibition of DHODH by TFN does not seem to cause depletion in neuronal activity. DHODH is associated with the mitochondrial respiratory chain, and according to Scialo et al., 2017, DHODH reduces ubiquinone in the process of the conversion of dihydroorotate during pyrimidine biosynthesis. Evidence shows that when ubiquinone becomes over-reduced, it can cause reverse electron transfers that are associated with excessive ROS production and causes Complex V to stop producing ATP [32]. Thus, DHODH-inhibition with TFN might affect mitochondrial respiratory chains/electron transport chains (ETC) and preserve neuronal functions. Moreover, we could not discount that these observations may be mediated by DHODH-independent effects of TFN, since it has been well established that TFN can inhibit different kinases at concentrations higher than 50 µM [20]. In our model, TFN does not cause any neuronal or tissue damage, however, based on the recent reports of existence of the de novo pathway for pyrimidine synthesis in the adult brain, we cannot exclude that in other contexts, TFN may affect neuronal cells by altering pyrimidine biosynthesis, [33].

Additionally, we observed that the amount of ATP decreases during oxidative stress, as already reported in the context of neuroinflammation [26,34]. ATP depletion could not be prevented by TFN in our set-up (Figure 2). However, diminished amounts of ATP do not appear to affect neuronal activity. This could mean that with TFN treatment, ATP synthesis has decreased either in response to acute stress or as a result of increasing mitochondrial movement. Another possible explanation could be that ATP consumption increased without negatively affecting the mitochondrial electron transport chain (ETC).

Furthermore, looking at the mitochondrial dynamics, we observed a reduction in mitochondrial length and area in acute hippocampal slices exposed to oxidative stress (Figure 3C,D), which is in line with our previous observations in peripheral root explants [28,30]. TFN reduced the length of mitochondria but not their area, implying changes in mitochondrial morphology, but not necessarily fragmentation. In consistent with this, a segregated morphological analysis of puncta, rod, network, and large mitochondria indicated an increase in networks rather than fragmentation (Figure 3C,D and Figure 6A–D). Thus, in consistent with our reports on peripheral root explants [28], TFN could prevent both oxidative stress-induced reductions in the mitochondrial area and fragmentation of mitochondria in acute brain slices. However, within the CNS, TFN promoted a reduction in mitochondrial displacement [28]. Interestingly, an increment of speed was observed during both oxidative stress alone and with TFN treatment along with oxidative stress (Figure 4A,B), suggesting the differences in mitochondrial dynamics in different tissue types.

To better understand which alterations of mitochondrial dynamics occur during the treatments, we categorized mitochondria in four different morphological categories. It is well established that, depending upon the cellular demand, mitochondria undergo fusion, fission, or biogenesis, and adopt a variety of forms or stages as part of mitochondrial homeostasis [35]. Mitochondrial shape could reflect different functional stages associated for instance with ATP generation, mitochondrial DNA segregation, calcium buffering, and the removal of damaged portions from mitochondria or mitophagy [36,37,38,39,40,41]. In this context, we observed a decrease in the length and area of rod-shaped and network mitochondria during oxidative stress (Figure 6A,C,E,G), suggesting that mitochondria are undergoing fragmentation, probably to eliminate depolarized mitochondria as a compensatory mechanism to increase the number of healthy functional mitochondria during stress situations [41]. On the other hand, the network mitochondria that are formed due to mitochondrial fusion [37,39,40] (Figure 5C,G) are important for buffering the damages, to get rid of the damaged portions, and distribution of functional portions [40,41]. The formation of large networks in response to stress was demonstrated in 2009 by Tondera et al. Importantly, the authors showed that this process of stress-induced mitochondrial hyperfusion (SIMH) is associated with an elevated production of mitochondrial ATP and represents an adaptive pro-survival response against stress [42]. Van der Bliek, 2009, further suggests that the fate of mitochondria is determined by the level of stress. While low stress might lead the mitochondria into SIMH, high and prolonged stress might promote the apoptotic fragmentation path [35]. Thus, TFN seems to reduce stress induced by H_2_O_2_ on neurons via DHODH-inhibition. The changes affecting network mitochondria may also lead to deficient ATP, also observed under oxidative stress, since, depending upon energy demand, mitochondria may form a network and get involved in ATP synthesis [36,38].

Additionally, during oxidative stress, the length and area increased for large mitochondria (Figure 6D,H), suggesting mitochondrial swelling. In general, large, and intensely fluorescent mitochondria indicate mitochondrial swelling and mitochondria undergoing mitophagy [37,39] (Figure 5D). Thus, the observed oxidative stress-mediated mitochondrial fragmentation and swelling within the CNS tissue suggests that mitochondria are unable to recover and do undergo degradation [37,39]. The addition of TFN during oxidative stress prevented oxidative stress-induced reduction in size of puncta-shaped mitochondria, and the reduction of both the length and size of network mitochondria (Figure 6B,F,G). It could be that TFN either prevented mitochondrial fission or promoted mitochondrial fusion to buffer the damages during oxidative stress. Moreover, the polarization of mitochondria seems to be maintained, as depicted by their ability to fuse [41] and form networks with TFN treatment. This is consistent with the findings by Miret-Casals et al. on mitochondrial fusion and elongation during DHODH-inhibition and limitation in the respiratory chain in proliferating cells [43].

Regarding mitochondrial motility, we observed during oxidative stress a significant reduction in the displacement of puncta-shaped mitochondria (Figure 7B), and an increment in the speed of rod-shaped, puncta-shaped and network mitochondria (Figure 7E–G), implying short distance mitochondrial transportation, in consistent with our observation in peripheral root explants [28]. However, oxidative stress induced comparatively faster mitochondrial movement with respect to the untreated slices (Figure 7E–H). This observation differs from the slow transport observed in peripheral root explants upon oxidative stress [28]. As we observed an increase in mitochondrial networks, the mitochondria that might fuse together to form networks might not need to travel longer and faster. TFN did not prevent these alterations but, apparently, induced an additional decrease in displacement and increase in the speed of puncta-shaped mitochondria, probably because they were forming the networks. Moreover, it has been reported that other factors, such as the depolymerization of cytoskeletal systems, may lead to impaired mitochondrial transportation. In this context, Zhang et al., 2018, demonstrated a H_2_O_2_- induced depolymerization of ß-actin filaments in neuronal cell lines incubated for 24 h with 200 µM H_2_O_2_ [44]. Such an effect is, however, very improbable in our experimental brain slices, which were incubated for only 30 min with H_2_O_2_.

Our study presents several limitations. Although the concentrations of H_2_O_2_ and TFN added to the brain sections were known, and in the case of H_2_O_2_ could be considered as unphysiological, the exact concentration of both substances at the depths at which we imaged the slices remained undetermined and are probably much lower. Moreover, the measurement of activity of DHODH in our experiment would have given more confirmative value to our existing results. Furthermore, we performed a two-dimensional analysis of the mitochondria, which may lead to errors in the estimations of shape and motility. However, since images under H_2_O_2_ stress and/or TFN conditions were corrected for time and compared to their corresponding baseline images, we consider that the errors introduced by the 2D analyses were minimized. An additional limitation is that, due to extremely high amounts of measured mitochondria, the semi-automated analysis could not be accurately confirmed by manual analysis. Additionally, we were unable to precisely determine mitochondrial boundary for the measurement using manual analysis, unlike in pixel-based measurements performed by the semi-automated analysis.

In summary, preventing the depletion of the oxidative metabolism and the alterations in mitochondrial motility might contribute to the preservation of neuronal firing in neuronal tissue treated with TFN during oxidative stress. We believe that the depletion of ATP does not necessarily mean energy failure but may reflect an enhanced consumption of ATP or slight shortcoming of ATP synthesis, while still maintaining the ETC as well as neuronal activity. In this context, as mentioned before, TFN may not negatively affect ETC when applied alone or together with hydrogen peroxide [45], but it seems to efficiently buffer mitochondrial damage, which could protect mitochondria against degradation. Thus, TFN seem to protect CNS tissue from oxidative damage. The protective effect seems to be mediated by its ability to prevent decrease in oxygen metabolism and neuronal activity, and its effect on mitochondrial dynamics.

## 4. Materials and Methods

### 4.1. Experimental Design and Settings

All the experiments were performed on the acute hippocampal slices of murine brain. The investigation of oxygen metabolism and synaptic transmission was performed simultaneously in an interface recording chamber with a continuous flow of carbogenated aCSF. After the initial baseline recording, the slices were treated with TFN and/or H_2_O_2_ and the second recordings were performed. For the analysis, after-treatment recordings were normalized to the baseline recordings. For the ATP assay, we treated acute hippocampal slices in continuously carbogenated HEPES aCSF. For the investigation of mitochondrial dynamics, we prepared acute hippocampal slices from mitoCFP mice brains and continuously supplied carbogenated HEPES aCSF to the slices. Mitochondrial images were taken using a two-photon microscope. Initial control images of mitochondria in the region of interest were captured. Then, the second images of the same region of interest were captured after the treatment. After-treatment analyses were normalized to the initial control images.

### 4.2. Experimental Animals and Ethics Statement

WT C57BL/6 mice were obtained from the Research Institute for Experimental Medicine—Forschungseinrichtungen für Experimentelle Medizin (FEM) at the Charité- Universitätsmedizin (Berlin, Germany). Breeding of transgenic Tg(Thy1-CFP/COX8A)S2Lich/J mice (*mitoCFP* mice) that express cyan fluorescence protein (CFP) in neuronal mitochondria was conducted at the FEM under specific pathogen-free conditions. All experimental procedures were conducted in strict accordance with Directive 2010/63/EU of the European Parliament and of the Council of 22 September 2010 and were approved by the Regional Animal Study Committee of Berlin, (LAGeSo-Landesamt für Gesundheit und Soziales Berlin), approvals ID: G0101-14 and T0002-10.

### 4.3. Preparation of Acute Hippocampal Sections

A quantity of 400 µm-thick acute brain slices were prepared based on the modified protocol from Nitsch et al., 2004 [29]. Hippocampal sections were obtained by slicing the 2/3rd of the caudal cerebral region. The intermediate hippocampal sections were used for the imaging experiments, as sections with comparable sizes could be generated from this region. Once the slices were obtained, the hippocampal region was dissected, preserving the hippocampal formation using the scalpel and the light microscope.

Brain slices were continuously supplied with carbogen (95% O_2_ and 5% CO_2_) in HEPES artificial cerebrospinal fluid (HEPES aCSF) (in mM: 92 NaCl, 2.5 KCl, 1.25 NaH_2_PO_4_, 30 NaHCO_3_, 20 HEPES, 25 Glucose, 5 Sodium ascorbate, 2 Thiourea, 3 Sodium pyruvate, 2 MgCl_2_, 2 CaCl_2_). The solutions were freshly prepared, and the pH adjusted between 7.3 and 7.4. The whole procedure was performed in temperatures between 34 and 37 °C.

To induce oxidative stress, acute slices were incubated for 30 min with hydrogen peroxide (H_2_O_2_) dissolved in aCSF. In our previous studies, 200 µM H_2_O_2_ induced mitochondrial damage [30], while 50 µM H_2_O_2_ induced only mitochondrial alterations [28]. Gülden et al., 2010, reported minimal cytotoxicity in cells cultured with 100 or 200 µM H_2_O_2_ for 1 h [46]. Hence, in our CNS model, we used 100 µM H_2_O_2_ for incubation in submerged chamber during mitochondrial imaging experiments to ensure diffusion of H_2_O_2_ inside the 400 µm-thick brain slices. Since, H_2_O_2_ concentrations are known to quickly decrease after short period of time [46,47], 200 µM H_2_O_2_ was used in the interface chamber during the electrophysiology experiments to ensure adequate H_2_O_2_ availability to the slices in air-water interface, unlike in submerged chamber where the slices are inside the solution. The other treatment groups were DMSO (0.0005%), H_2_O_2_ (100 µM) +DMSO (0.0005%), and H_2_O_2_ (100 µM), along with TFN (50 µM) and TFN (50 µM) alone. DMSO was used as a vehicle control for TFN.

### 4.4. Simultaneous Electrophysiology and Oxygen Partial Pressure (pO_2_) Recordings in Hippocampal Slices

Hippocampal slices (400 µm thickness) were prepared using a Leica VT1200 S vibratome (Leica, Wetzlar, Germany), as mentioned above. The slices were stored in an interface chamber and continuously supplied with freshly prepared and carbogenated aCSF (in mM: 129 NaCl, 21 NaHCO_3_, 10 Glucose, 3 KCl, 1.25 NaH_2_PO_4_, 1.6 CaCl_2_, and 1.8 MgCl_2_) for at least 90 min before the investigation (temperature ~34 °C). To induce oxidative stress, 200 µM H_2_O_2_ was added to the bath solution for 30 min.

Stimulus-induced population spikes (PS) and pO_2_ were recorded using a glass microelectrode filled with 154 mM NaCl and a Clark-style oxygen electrode (tip diameter: 10 µm; Unisense, Aarhus, Denmark), respectively. Both electrodes were placed in the stratum pyramidale of the CA1 area while stimulation (2 pulses of 100 µs duration with a 50 ms interval every minute) was performed with a stimulation electrode placed in the stratum radiatum between CA3 and CA1 using a Master 8 system (A.M.P.I., Jerusalem, Israel). To measure changes in neuronal respiration, the pO_2_ electrode was advanced in 20 µm steps through the slices using a calibrated micromanipulator (Narishige, Tokyo, Japan). In the in vitro condition, pO_2_ distribution depends on cellular O_2_-consumption since O_2_ diffusion and solubility are constant [48]. Typically, a vertical O_2_-gradient can be measured, from the surface till the core of the slice, the depth in which pO_2_ ceases to decrease. For electrophysiology measurements, baseline measurements were performed at 100 µm below the tissue surface for 15 min before and after 30 min treatment with H_2_O_2_ and H_2_O_2_ + TFN. Analog signals were digitalized using a Power1401 and recorded using Spike 2 software (Cambridge Electronic Design Limited, Cambridge, UK) for the recording. Offline analysis of the data was performed using Spike2.

### 4.5. Adenosine Triphosphate (ATP) Assay

After the treatment of the slices, ATP assay was performed using the Abcam ATP assay kit (Abcam, ab83355) according to the manufacture’s protocol. Briefly, the tissue was homogenized in ATP assay buffer and centrifuged at 13,000× *g* for 5 min. Then, the supernatant was collected and deproteinization with perchloric acid (PCA) was performed. ATP standards, in the range of 0 to 1 nmol, as well as samples, were then incubated with reaction-mix for 30 min. The fluorometric detection was performed at 535/587 nm in a plate reader (GloMax^®^-Multi Detection System, Promega Corporation, Madison, WI, USA). The calculation of the amount of the ATP in each slice was performed after the subtraction of the sample background and with the multiplication of the dilution factor, as described in the protocol by the manufacture.

### 4.6. Mitochondrial Imaging Using Two-Photon Laser Scanning Microscope in Acute Hippocampus Sections

To image mitochondria in slices prepared from the mitoCFP transgenic mouse (Tg(Thy1-CFP/COX8A)S2Lich/J), an 830nm excitation wavelength and photomultiplier at 525 nm with 50 nm bandwidth was selected.

At first, a region of interest (ROI) was selected and a time-lapse image stack of 30 steps with intervals of 3.6 s was obtained. Then, aCSF along with treatment was added without moving the objective or the holding chamber. After 30 min, the same region was imaged again with same setting. This procedure was repeated with each single treatment group.

### 4.7. Image Analysis of Mitochondrial Morphology and Dynamics in Acute Hippocampal Slices

Huygens deconvolution Scientific Volume Imaging software (SVI, The Netherlands) was applied for the restoration of mitochondrial images obtained with two-photon microscopy. Images were opened in Huygens professional X11 and enabled automatic computation of point spread function (PSF). Then, the images were inspected with the logarithmic vertical mapping function. Once, the PSF was computed, the Classic Maximum Likelihood Estimation (CMLE) algorithm was applied to obtain effectively deconvolved images.

Images were corrected for the background noise and image movement using Fiji Is Just ImageJ (Fiji) software [49,50]. The correction of the movement of the images was conducted using StackReg plugin [51]. After the preprocessing of the images, segmentation of mitochondria was preformed using Trainable Weka segmentation plugin in Image J software. All the automation for the analysis was controlled and supervised by the experimenter.

For morphology analysis, the corrected images were first segmented with Trainable Weka segmentation plugin in Image J software [52]. A few mitochondria and backgrounds were selected in each image for the plugin to train itself. However, the segmentation output was not satisfactory; subsequently, the plugin was given more examples of mitochondria and backgrounds. Then, the probability map of the segmented mitochondria was used for the analysis with Volocity 6.3 software (Perkin Elmer, Rodgau, Germany). In Volocity software, the mitochondria were identified based on the fluorescence intensity threshold against the background and noises, and the measurements for the length and area of the selected objects were extracted.

For motility analysis, from the corrected images, five random ROIs within the image were selected. Then, each ROI was separately processed into Track mate plugin [53] for tracking mitochondrial transport (Supplementary Videos S1 and S2. The measurements of mitochondrial speed and displacement were extracted for further analysis).

### 4.8. Statistical Analysis

The data were analyzed with the Prism software (GraphPad, San Diego, CA, USA). First, the Gaussian distribution of the variables was confirmed using all the three normality tests— KS, D’Agostino and Pearson omnibus, and Shapiro–Wilk. Non-normally distributed data were treated as non-parametric. Comparisons between the two groups were performed by *t*-test for the normal parametric data. Comparisons between two groups of non-parametric data were performed by Mann–Whitney U test. Comparison between more than two groups were analyzed with one-way analysis of variance and with Kruskal–Wallis test for non-parametric data followed by Dunn’s post hoc test. All the data are shown as mean ± SD. The data are shown as Tukey boxplot where the central line denotes the median; the lower and upper boundaries denote the 1st and 3rd quartile, and the whiskers denote the data except the outliers presented as individual dots. The outliers were not included in the analysis. *p* values ≤ 0.05 were considered significant. The significance of the data was further depicted as * *p* ≤ 0.05, ** *p* ≤ 0.01, *** *p* ≤ 0.001, and **** *p* ≤ 0.0001.

## 5. Conclusions

Here, we conclude that TFN contributes to mitochondrial protection during oxidative stress. The drug might influence mitochondrial bioenergetics by reversibly limiting the reduction of flavin mononucleotide (FMN) via DHODH and thus, electron transport in the ETC [54]. This in turn reduces ROS production, as ETC is one of the major ROS producers, while the intracellular antioxidants work against the extracellular ROS. Additionally, due to the presence of multiple electron donor complexes, the ETC is not completely shut off. Thus, TFN does not significantly depress the ATP synthesis and helps maintain mitochondrial dynamics and neuronal activity.

## Figures and Tables

**Table 1:** Mitochondrial length in acute hippocampal slices.

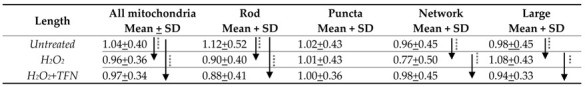

*** *p* ≤ 0.001, **** *p* ≤ 0.0001.

**Table 2:** Mitochondrial area in acute hippocampal slices.

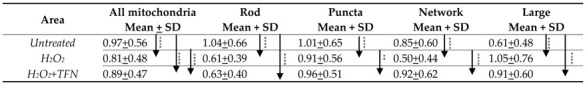

** *p* ≤ 0.01, **** *p* ≤ 0.0001.

**Table 3:** Mitochondrial displacement in acute hippocampal slices.

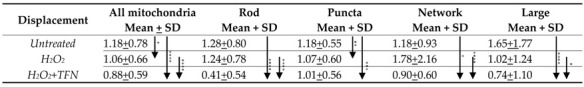

* *p* ≤ 0.05, ** *p* ≤ 0.01, *** *p* ≤ 0.001, and **** *p* ≤ 0.0001.

**Table 4:** Mitochondrial speed in acute hippocampal slices.

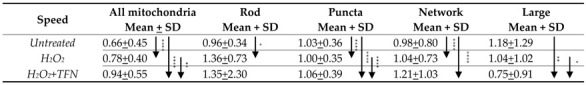

* *p* ≤ 0.05, ** *p* ≤ 0.01, **** *p* ≤ 0.0001.

## Data Availability

The datasets during and/or analyzed during the current study available from the corresponding author on reasonable request.

## Supplementary Materials

The following supporting information can be downloaded at: https://www.mdpi.com/article/10.3390/ijms23031538/s1.
